# Impact of chronic hyperglycaemia on the coronary microcirculation – benefits of targeting IL-36 and diet reversal

**DOI:** 10.1007/s00395-025-01107-y

**Published:** 2025-04-17

**Authors:** Juma El-Awaisi, Dean Kavanagh, Silke Heising, Ina Maria Schiessl, Simon J. Cleary, David J. Hodson, Neena Kalia

**Affiliations:** 1 Microcirculation Research Group, Department of Cardiovascular Sciences, School of Medical Sciences, College of Medicine and Health, University of Birmingham, Birmingham, B15 2 TT UK; 2 Department of Metabolism and Systems Research, School of Medical Sciences, College of Medicine and Health, University of Birmingham, Birmingham, B15 2 TT UK; 3 Department of Biomedicine, Aarhus University, Aarhus, Denmark; 4 Institute of Pharmaceutical Science, King’s College London, London, UK; 5 Oxford Centre for Diabetes, Endocrinology and Metabolism, Radcliffe Department of Medicine, University of Oxford, Oxford, UK

**Keywords:** Coronary microcirculation, Ischaemia–reperfusion injury, Interleukin- 36, Hyperglycaemia, Inflammation

## Abstract

Following myocardial infarction (MI), patients with type 2 diabetes mellitus (T2DM) have poorer prognosis which may be linked to increased susceptibility of coronary microvessels to injury. Interleukin-36 (IL-36) may mediate this injury but its role in the microcirculation of the chronically hyperglycaemic injured heart is unknown. Intravital and laser speckle imaging of the anaesthetised mouse beating heart evaluated the impact of a 16-week high fat diet (HFD)-induced hyperglycaemia ± myocardial ischaemia–reperfusion injury (IR) injury on coronary microvessels. Neutrophil/platelet recruitment, neutrophil extracellular trap formation, cellular necrosis, vascular leakage, vascular tonal changes, functional capillary density, overall ventricular perfusion and levels of circulating inflammatory cytokines were assessed alongside the vasculoprotective ability of an IL-36 receptor antagonist (IL-36Ra). Whether heightened microvessel damage in injured HFD mice was permanent or reversible was investigated after normalising hyperglycaemia through diet reversal (DR). Microcirculatory events assessed were perturbed basally in HFD mice and further after injury. IL-36Ra mitigated these effects and improved infarct size. DR was also beneficial, decreasing neutrophil recruitment to levels below those seen in untreated mice. Mechanistically, benefits of both IL-36Ra and DR could be explained by decreased endothelial oxidative stress and VCAM-1 expression and possibly by raised levels of IL-4/IL-13. Basal changes in chronically hyperglycaemic coronary microvessels that are heightened in the aftermath of reperfusion may explain the poorer outcomes in MI patients with T2DM. These findings are the first to highlight the specific benefits of IL-36 inhibition and reversing hyperglycaemia through dietary modification on the coronary microcirculation in a preclinical model of T2DM.

**Supplementary Information:**

The online version contains supplementary material available at 10.1007/s00395-025-01107-y.

## Introduction

Type 2 diabetes mellitus (T2DM), associated with obesity, is a common comorbidity in cardiovascular diseases (CVDs), with myocardial infarction (MI) accounting for up to 75% of the deaths in these patients [1]. It affects 25–30% of patients undergoing flow restoring primary percutaneous coronary intervention (PCI) [42]. Despite successful PCI, T2DM remains a strong predictor of short and long-term adverse effects, including larger infarct size, longer hospital stays, increased likelihood of developing heart failure and/or higher mortality [17, 34]. This is likely due to the increased susceptibility of the diabetic coronary microcirculation to the ischaemia–reperfusion (IR) injury that occurs post-PCI [21, 29, 36]. Increasing recognition that the coronary microcirculation is a primary target of IR injury has refocussed efforts on identifying vasculoprotective strategies [22, 29, 46, 55, 58]. Whilst cardiovascular macrovascular and non-cardiovascular microvascular complications are well recognised in T2DM, little is known about how hyperglycaemia disturbs coronary microvessels in vivo due to an inability to resolve them using current clinical imaging tools.

Novel therapies are required to protect the delicate coronary microcirculation against the detrimental effects of acute IR injury. Moreover, such therapies need to remain effective in the diabetic heart to improve outcomes in these patients. Interleukin- 36 (IL‐36) belongs to the IL‐1 family and includes three agonists (IL‐36α/β/γ) and a natural IL‐36 receptor antagonist (IL‐36Ra). Members of this family are frequently the first and most upstream to be produced in response to injury and therefore considered good targets for therapeutic intervention [52]. IL-36 not only amplifies the effects of IL-1, but is also a potent mediator of inflammation in its own right, triggering secretion of other pro-inflammatory cytokines, including TNFα, IL-1β, IL-6 and IL-8, from diverse cell types [18]. Although it is well established that the IL-36/IL-36R pathway is highly inflammatory in the skin and lungs, we are still at an early stage in our current understanding of its role in CVDs. Our recent intravital imaging of the mouse beating heart showed for the first time that IL-36 inhibition prevented multiple coronary microcirculatory disturbances and improved ventricular perfusion post-IR injury [10, 12]. Notably, recent studies identified higher circulating levels of IL-36, and decreased IL-36Ra, in patients with obesity-associated T2DM [15, 37]. Collectively, these studies suggest that targeting IL-36 post-IR injury may protect the vulnerable coronary microcirculation in the setting of chronic hyperglycaemia.

Growing evidence suggests that lifestyle changes that involve improving diet and losing weight can lead to T2DM remission [30, 54]. It remains to be established, however, whether reversing diet-associated diabetes can protect the coronary microvessels from potentially heightened damage in the event of an MI. It has been shown that hearts from mice switched from a diet high in fat to a normal chow diet were more resistant to reperfusion injury as evidenced by less cardiac catalase expression and mitochondrial damage [38]. However, little is known about the impact of reversing chronic hyperglycaemia through diet modification on the IR injured coronary microcirculation.

We therefore combined intravital and laser speckle contrast (LSCI) imaging of the anaesthetised mouse beating heart to assess whether diet-induced chronic hyperglycaemia increased the susceptibility of the coronary microvasculature to reperfusion injury and whether any microvasculature perturbations could be reversed with either IL-36 antagonism or diet reversal.

## Methods

### Mouse diets and myocardial IR injury

Experiments were conducted on male C57BL/6 mice (2–4 months). All procedures received local approval from the Animal Welfare and Ethical Review Body (AWERB) and were conducted in accordance with the Animals (Scientific Procedures) Act of 1986 (HO Project licence P552D4447; conforms to the ethical standards laid down in the 1964 Declaration of Helsinki and its later amendments). Mice were fed either a normal chow diet (ND) or a high fat diet (HFD; 60% kcal from fat; D12492; Research Diets) for 16 weeks. The latter closely mimics the progression of T2DM in humans, leading to the development of obesity, hyperglycaemia and increased insulin secretion and resistance [7]. A separate group of mice, fed a HFD for 16 weeks, had their diet reversed (DR) back to normal chow for 8 weeks and were compared, where appropriate, to age-matched mice fed a ND for 24 weeks. Plasma glucose levels were measured via tail tip vein puncture using a Contour XT glucometer (Bayer). Anaesthesia was induced using intraperitoneal ketamine hydrochloride (100 mg/kg) and medetomidine hydrochloride (10 mg/kg). Mice were intubated and ventilated with medical oxygen using a MiniVent rodent ventilator (stroke volume: 220 μl, respiratory rate: 130 pm; Harvard Apparatus). The carotid artery was cannulated to facilitate infusion of antibodies, dyes, saline and IL-36Ra. Myocardial IR injury was performed as previously described [4, 10, 28]. Briefly, the left anterior descending (LAD) artery was suture ligated for 45 min, followed by reperfusion for either 2 h for tissue analysis, 2.5 h for intravital observations, and 4 h for infarct measurement. For some studies, recombinant mouse IL-36Ra (15 μg/mouse; Novus Biologicals) was injected intra-arterially at 10 min pre-reperfusion and 60 min post-reperfusion [10]. At the end of experiments, mice were killed by cervical dislocation for tissue and plasma collection.

### Intravital and laser speckle contrast (LSCI) imaging

**Fig. 1:**
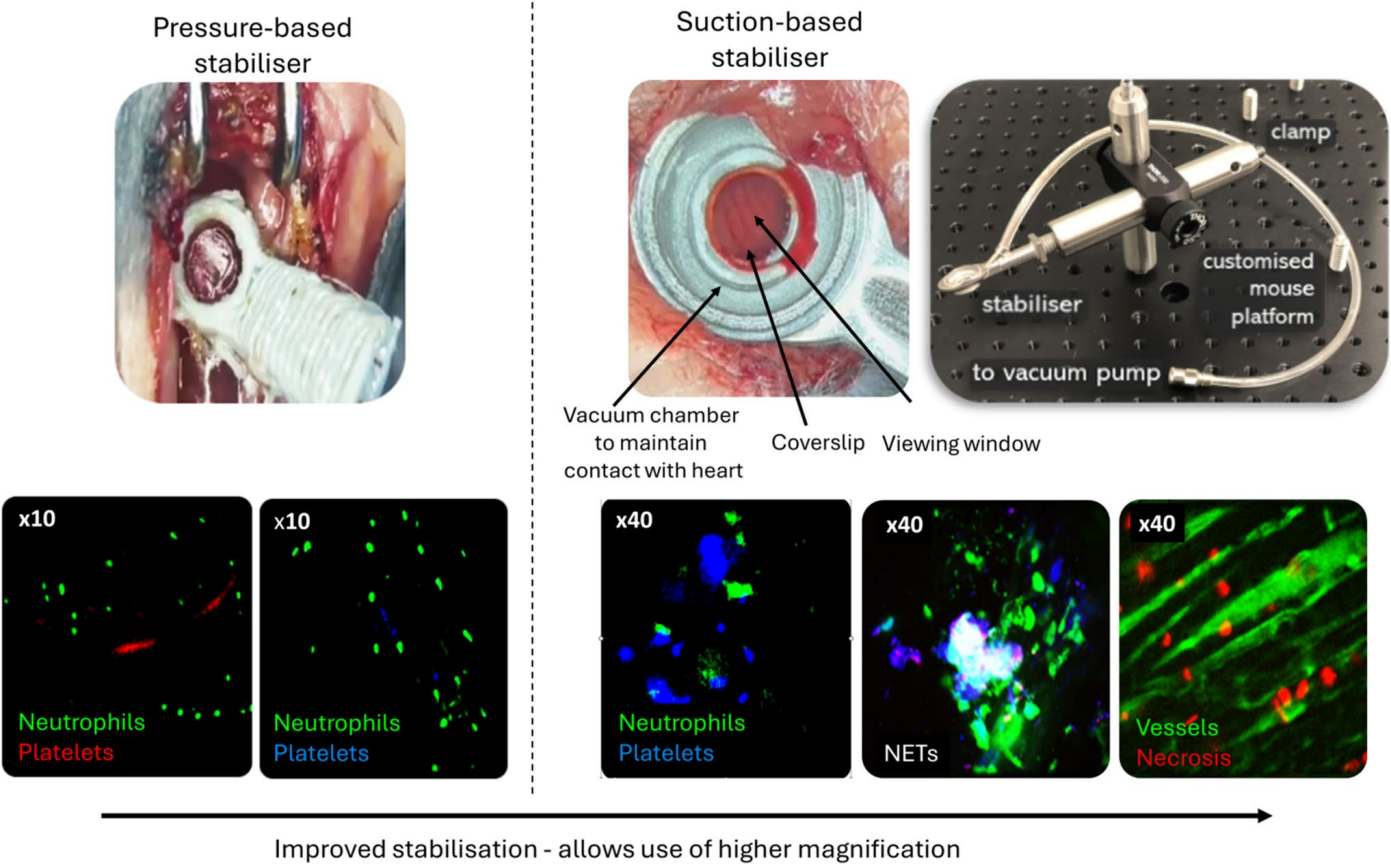
Real-time intravital imaging of the anaesthetised mouse beating heart was performed by attaching a custom-designed 3D printed stabilizer ring using gentle pressure to the LV downstream of the ligation site. This allowed simultaneous imaging of neutrophils and platelets in the coronary microcirculation. This stabilisation procedure was optimised using gentle negative suction pressure to attach a custom imaging window to the LV to enable higher magnification imaging of cellular necrosis and NETs

Real-time intravital imaging of the anaesthetised mouse beating heart was performed as previously described [4, 10, 12, 28]. Briefly, a custom-designed 3D printed stabilizer ring was placed using gentle pressure on the left ventricle (LV) downstream of the ligation site (Fig. 1). To simultaneously image neutrophils and platelets, 20 μL of a PE anti-mouse Ly- 6G antibody (BioLegend) and an APC anti-mouse CD41 antibody (BioLegend) were injected intra-arterially. Fluorescein isothiocyanate conjugated to bovine serum albumin (FITC-BSA; Sigma) was injected to qualitatively assess overall vascular perfusion and functional capillary density (FCD). The ability of IL-36 cytokine to directly mediate neutrophil and/or platelet adhesion was assessed by topically applying recombinant mouse IL-36γ (20 μL at 200 ng/mL; R&D Systems) or PBS for 15 min. This was replaced with fresh cytokine/PBS for 15 min and the process was repeated 10 times for a total duration of 2 h.

IL-36 cytokines are classed as *bona fide* damage associated molecular patterns (DAMPs), released as inactive precursors after cellular necrosis [44, 60]. Their pro-inflammatory biological activity is amplified almost 1000-fold by neutrophil proteases, specifically elastase and cathepsin G, released in soluble form from activated neutrophils or bound to the surface of expelled neutrophil extracellular traps (NETs) [5]. Considering this, we optimised the intravital technique to enable higher magnification imaging of cellular necrosis and NETs*,* something not previously performed in the beating heart. However, heart stabilisation using a stabiliser gently pressed onto the heart, whilst effective, did not reduce motion sufficiently enough to permit the higher magnification imaging needed to assess changes in vessel tone, NETs or cellular necrosis in vivo. Therefore, we developed an approach, adapted from previous methods for stabilising lungs, using gentle negative pressure to suction attach a custom imaging window to the LV (Fig. 1) [6, 41]. Vascular leakage of FITC-BSA in response to histamine was then assessed by topically applying histamine (30 μM; R&D Systems) within the centre of the stabilizer ring for 15 min. Changes in the diameter of a medium sized blood vessel in response to topical application of the vasoconstrictor phenylephrine (PE; 0.3 μM; Sigma), and subsequent vasodilation of the same vessel in response to topical acetylcholine (ACh; 10 μM; Bio-techne), was also assessed intravitally. To intravitally image NETs, a triple-labelling approach was used to visualize co-localization of three key NET components, namely extracellular DNA, histones (H2 A.X) and neutrophil elastase. A SYTOX™ green nucleic acid stain (Thermo Fisher), a PE H2 A.X phospho antibody (BioLegend) and an Alexa Fluor 647 neutrophil elastase antibody (Santa Cruz) were injected intra-arterially [32]. In separate mice, propidium iodide (PI; Thermo Fisher) was injected to visualise cellular necrosis [2]. This dye does not permeate intact cell membranes and therefore labels necrotic cell nuclei.

Intravital imaging was performed using a microscope (BX61 WI, Olympus) equipped with a Nipkow spinning disk confocal head (Yokogawa CSU) and an Evolve EMCCD camera (Photometrics). Images were captured, stored and analysed using Slidebook 6 software (Intelligent Imaging Innovations, USA). To aid analysis, captured videos were subjected to post-acquisition image repair using an in-house software (*Tify*) in which out-of-focus frames were removed [27]. Neutrophils, platelet aggregates, leakage and necrotic cells were quantitated by placing a mask around PE-Ly6G^+^, APC-CD41^+^, FITC-BSA^+^ and PI^+^ areas respectively. Integrated fluorescence density, which took into account size and flourescence intensity, was calculated using ImageJ (NIH). NETs were quantified by placing a mask around PE-H2 A.X^+^, A647-NE^+^, and SYTOX^+^ areas and the area of co-localisation was calculated using ImageJ (NIH).

LSCI was positioned above the exposed heart to quantitate overall LV myocardial perfusion in beating hearts (moorFLPI- 2; Moor Instruments) [11, 28]. A demarked area, downstream of the ligation site, was identified for collection of flux (perfusion) data during pre-ischaemia, ischaemia and at every 15 min post-reperfusion. 1000 frames were captured at each time point using the manufacturer supplied image software (mFLPI2Measure V2.0; mFLPIReview V5.0) at a frame rate of 25 Hz and using spatial processing (sliding window, time constant: 0.1 s). Basic Speckle Analysis software (SpAn), written in-house, allowed identification and collation of flux values during diastole for each time point [11].

### Immunostaining and flow cytometric analysis of IL-36, IL-36R, VCAM-1 and oxidative damage

Frozen heart tissue Sects. (10 µm) were incubated with primary antibodies against IL-36R, IL-36α/β or IgG control antibodies (1:100 dilution; R&D Systems) and a donkey anti-goat Alexa Fluor- 647 secondary antibody (1:100; Abcam). Sections were also incubated with a PE anti-mouse CD31 antibody (1:100; BioLegend). Images were captured using an EVOS FL microscope (Thermos Fisher Scientific) and ImageJ was used to quantify mean fluorescence intensity (MFI). Flow cytometry analysed IL-36R expression and oxidative damage on endothelial cells (ECs) and cardiomyocytes (CMs) [10]. Briefly, hearts were 0.1% collagenase-digested to obtain single-cell suspensions and then incubated with an anti-IL-36R primary antibody (1:100; R&D Systems), Alexa- 647 secondary antibody (1:100; Biolegend), anti-DNA/RNA oxidative damage antibody (1:100 dilution; Abcam), anti-CD31 antibody (1:100; Biolegend), anti-cTnT antibody (1:100; Miltenyi Biotec), Alexa Fluor- 647 anti-mouse VCAM- 1 antibody (BioLegend), and a zombie dye (1:500 dilution, BioLegend), along with appropriate IgG controls. The DNA/RNA oxidative damage antibody binds with high specificity and affinity to various products of oxidative DNA damage induced by reactive oxygen species (ROS), including 8-hydroxy- 2′-deoxyguanosine (8-OHdG). 8-OHdG is one of the most widely recognised and validated biomarkers of oxidative DNA damage. For each sample, 250,000 events were analysed (CyAn™ ADP and Summit 4.3 software; Beckman Coulter).

### Myocardial infarct size analysis

The LAD artery was re-ligated 4 h after reperfusion and 0.5% Evans blue dye (Sigma) was infused via the carotid artery to delineate the area at risk (AAR). The mouse was then killed, and the harvested heart was sliced and incubated with 2,3,5-triphenyltetrazolium chloride (TTC; Sigma). Sections were imaged using a stereomicroscope and analysis was performed using ImageJ to quantitate the infarct size (TTC^neg^ white regions) as a percentage of the AAR (TTC^pos^ red regions/Evans blue^neg^ regions).

### Multiplex immunoassay to assess circulating cytokine levels

A commercial multiplex immunoassay was used to investigate serum cytokine levels of 23 inflammatory cytokines. Anti-coagulated blood was collected at 2 h post-reperfusion and centrifuged (2000 rpm; 10 min) to obtain serum. Samples were then loaded onto a BIO RAD® Bio-Plex Pro mouse cytokine 23-plex immunoassay plate (Bio-Rad Laboratories) and analysed using a Luminex 200 plate reader.

### Statistical analysis

All statistical analysis was performed using GraphPad 9.0 software (GraphPad Software Inc., USA). Multiple comparisons between three or more groups were performed using a one-way ANOVA followed by a Tukey post-hoc test. Results are presented as mean ± SEM, with *p* < 0.05 considered statistically significant. LSCI results cannot be expressed in absolute perfusion values and were therefore expressed as arbitrary perfusion flux units and as a percentage of baseline.

## Results

### HFD increases weight and plasma glucose and disturbs vascular leakage and vascular tone

**Fig. 2:**
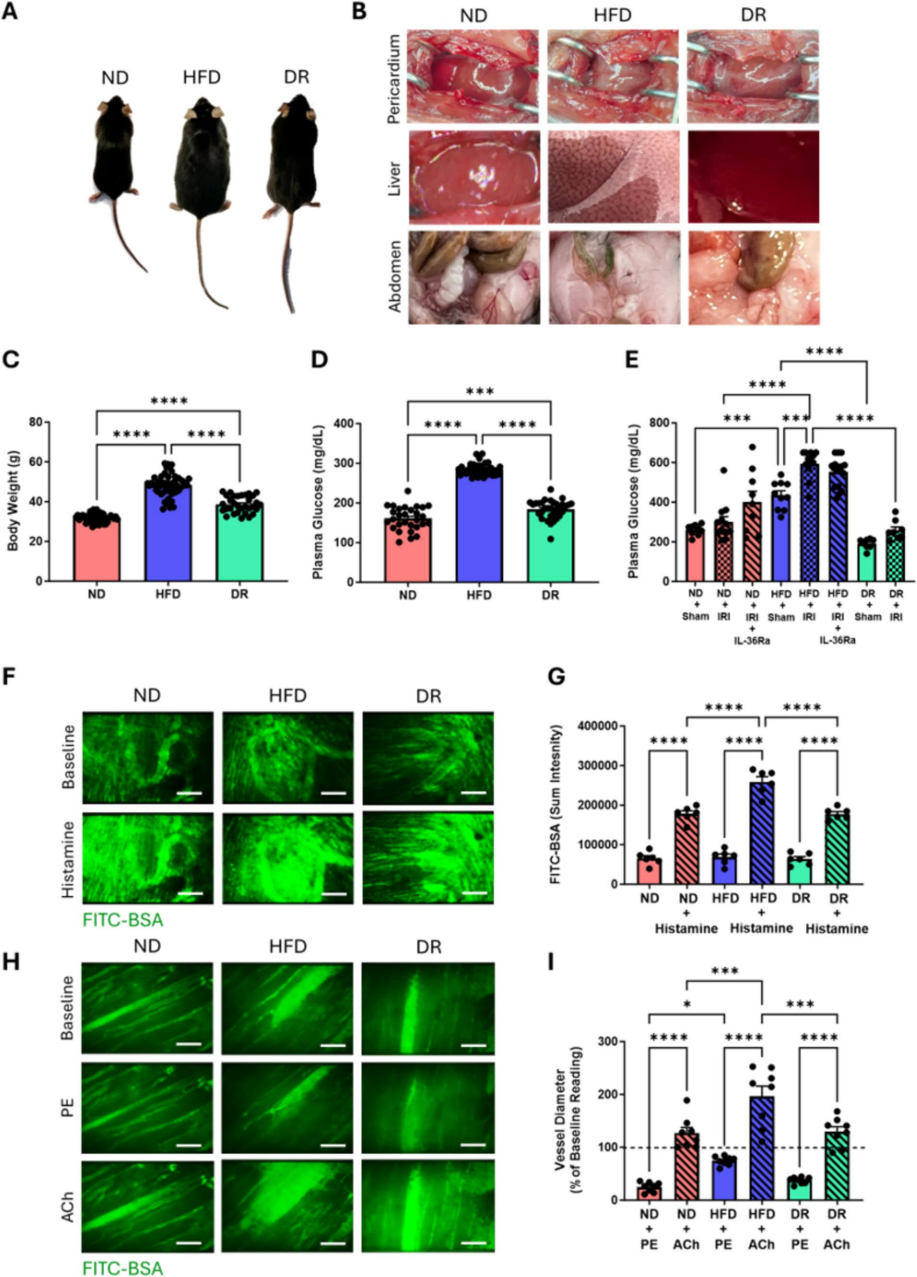
Impact of a HFD on body weight, plasma glucose, vascular leakage and vascular tone in vivo. **A** Representative images of ND, HFD and DR mice. **B** Representative images showing increased fat within pericardium, abdomen and liver of HFD mice. **C** Quantitative analysis of mouse weights at time of experimentation. N = 32–49/group. **D** Quantitative analysis of basal plasma glucose levels. N = 29–39/group. **E** Quantitative analysis of plasma glucose levels after IR injury. N = 8–17/group. **F** Representative intravital images of healthy beating heart showing FITC-BSA leakage from blood vessels before and after topical histamine (30μM) application. n = 6 areas analysed from N = 3/group. **G** Quantitative analysis of intravital data for FITC-BSA leakage. **H** Representative intravital images of healthy beating heart showing blood vessels before and after topical phenylephrine (PE; 0.3μM) and acetylcholine (ACh; 10μM) application. **I** Quantitative analysis of intravital data for vascular tone. n = 8 vessels analysed from N = 3/group. *p < 0.05, ***p < 0.001, ****p < 0.0001 when tested using a one-way ANOVA followed by a Tukey multiple comparison test. *ND* normal diet, *HFD* high fat diet, *DR* diet reversal. Scale bar: 100 μm (**F**); 50 μm (**H**)

Body weight increased significantly in HFD mice compared to ND mice, with fat macroscopically visible in the pericardium, liver and abdomen. Body weight of DR mice was significantly lower than HFD mice but remained higher than ND mice (Fig. 2a–c). Non-fasted plasma glucose levels increased significantly in HFD mice compared to ND mice, indicative of hyperglycaemia. Glucose levels were significantly reduced in DR mice compared to HFD mice but remained elevated compared to ND mice (Fig. 2d). Plasma glucose levels spiked further in response to myocardial IR injury in all mice, but this only attained statistical significance in injured HFD mice (Fig. 2e).

Vascular integrity and vasotone was assessed intravitally. No basal difference in FITC-BSA leakage was observed between all mice, with FITC-BSA contained within blood vessels indicating preserved vascular integrity. However, macromolecular leakage increased in response to topical histamine, primarily affecting the delicate coronary capillaries, but was significantly higher in HFD mice compared to both ND and DR histamine treated mic. (Fig. 2f, g). The patchy, irregularly shaped FITC-BSA fluorescent flares, which were not aligned with the parallel directionality of the capillaries, was typical of leakage rather than vessel calibre changes in response to histamine. Indeed, when the vasculature was not obscured by this leak, any medium-sized vessels in the field of view did not appear to change diameter during the imaging period. Topical PE reduced the diameter of medium sized coronary vessels in all mice. This vasoconstriction response was significantly reduced in HFD mice compared to ND mice. Topical ACh significantly increased the diameter of the same PE treated coronary vessel in all mice but this vasodilatory response was significantly greater in HFD mice (Fig. 2h, i).

### HFD increases cardiac IL-36R, IL-36α and IL-36β expression—alleviated with DR

**Fig. 3:**
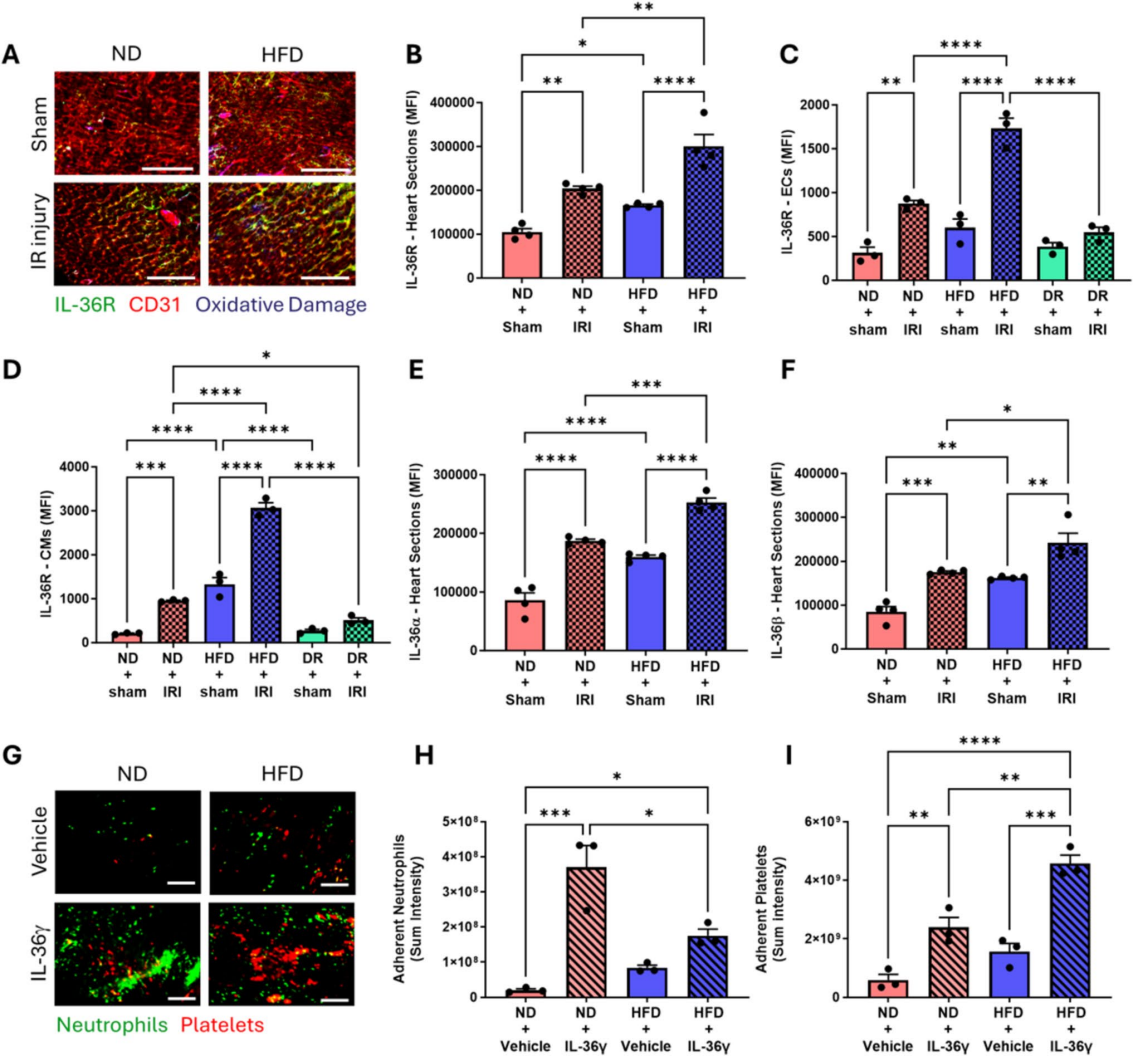
Impact of a HFD on cardiac IL-36R/α/β expression and response of coronary microcirculation to IL-36 in vivo. **A** Representative immunofluorescence images of frozen heart sections to show IL-36R, endothelial cells (ECs; CD31) and oxidative damage expression (not analysed but increased expression in injured HFD mice). **B** Quantitative analysis of immunofluorescence images for IL-36R expression. N = 4/group. Flow cytometric analysis for IL-36R expression on **C** ECs and **D** cardiomyocytes (CMs). N = 3/group. Quantitative analysis of immunofluorescence images (not shown) for **E** IL-36α and **F** IL-36β expression. N = 4 mice/group. **G** Representative intravital images of the healthy beating heart after topical exposure to IL-36γ (100 ng/ml). Images show neutrophils and platelets at 90 min post-treatment. Quantitative analysis of intravital data for adherent **H** neutrophils and **I** platelets at 120 min post-exposure to IL-36γ. N = 3/group. *p < 0.05, **p < 0.01, ***p < 0.001, ****p < 0.0001 when tested using a one-way ANOVA followed by a Tukey multiple comparison test. *ND* normal diet; *HFD* high fat diet. Scale bar: 100 μm

Immunostaining demonstrated that basal IL-36R expression increased significantly in HFD sham mice compared to ND sham mice. This increased further with IR injury in ND and HFD mice, with the greatest expression identified in the latter (Fig. 3a, b). This data was confirmed using flow cytometry where a similar pattern of IL-36R expression was observed on both coronary ECs and cardiomyocytes. Significantly reduced expression was observed in injured DR mice compared to injured HFD mice (Fig. 3c, d). Immunostaining for IL-36α/β demonstrated a similar pattern of expression to IL-36R (Fig. 3e, f). The functional activity of IL-36R was investigated intravitally in response to topical IL-36γ. Rapid neutrophil and platelet aggregate presence was observed in ND and HFD mice, which remained significantly elevated, after IL-36γ exposure (Figs. 3g–I and Figure S1).

### HFD increases thromboinflammatory disturbances, NETs and cellular necrosis – alleviated with IL-36Ra and DR

**Fig. 4:**
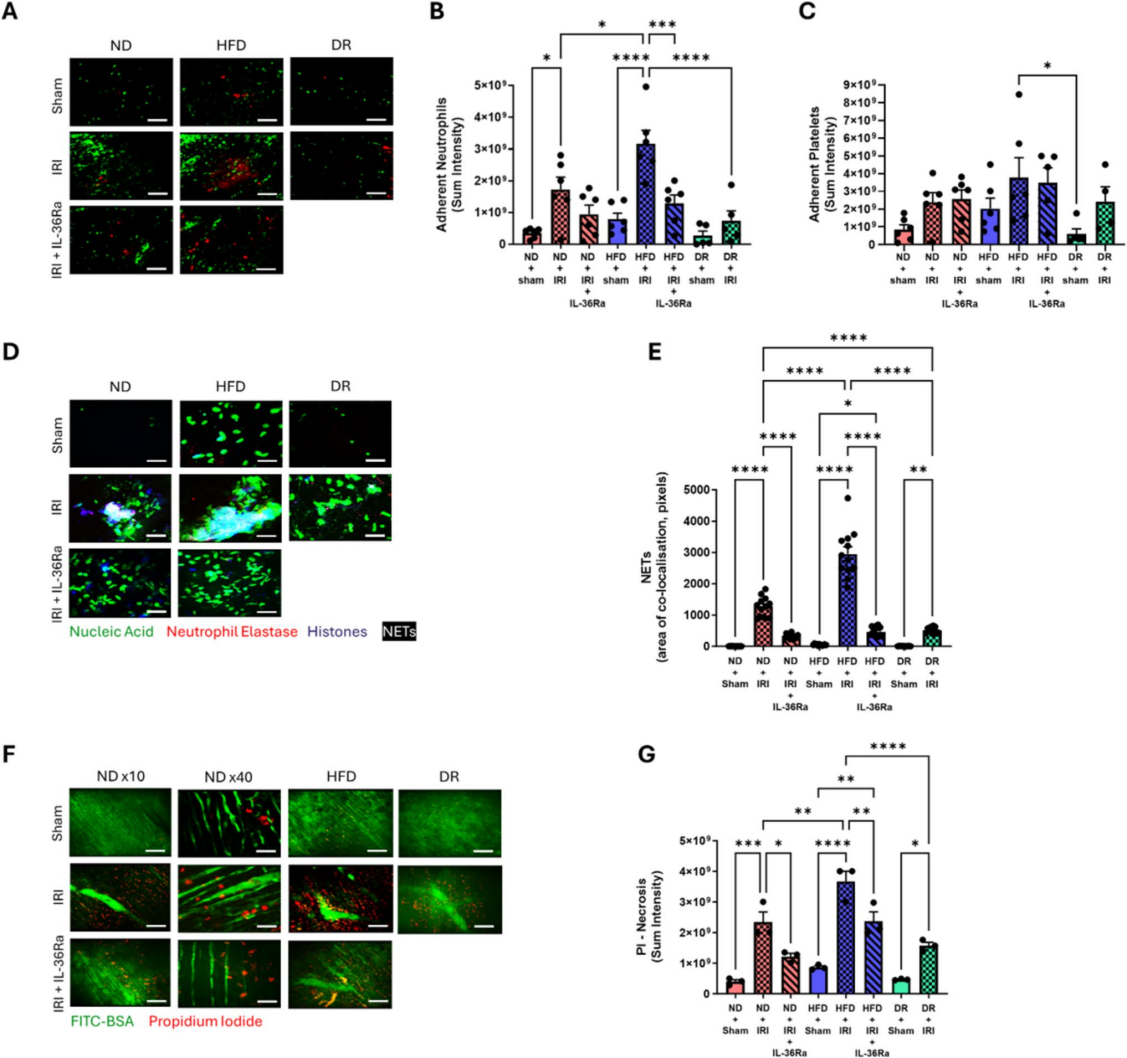
Impact of a HFD, IL-36Ra therapy and diet reversal on thromboinflammatory disturbances, NET formation and cellular necrosis in vivo. **A** Representative intravital images showing neutrophils and platelets at 120 min post-reperfusion in mice. Quantitative analysis of intravital data for adherent **B** neutrophils and **C** platelets at 120 min post-reperfusion. N = 5–6/group. **D** Representative intravital images showing NETs (merged white areas) at 120 min post-reperfusion. **E** Quantitative analysis of intravital data for NETs. n = 12 areas analysed in N = 3/group. **F** Representative intravital images showing cellular necrosis identified by propidium iodide (PI) labelling at 120 min post-reperfusion. **G** Quantitative analysis of intravital data for cellular necrosis. N = 3/group. Scale bar: *p < 0.05, **p < 0.01, ***p < 0.001, ****p < 0.0001 when tested using a one-way ANOVA followed by a Tukey multiple comparison test. *ND* normal diet, *HFD* high fat diet, *DR* diet reversal. Scale bar: 100 μm (**A**), 25 μm (**G**); 100 μm (**I**); 25 μm (I: NDx40)

No significant basal difference in adherent neutrophils was observed intravitally between sham ND and sham HFD mice but was higher in the latter. However, neutrophil adhesion, occurring mainly within coronary capillaries, significantly increased in ND and HFD mice in response to injury but more so in the latter group. Indeed, neutrophils often formed clusters, in HFD mice making it difficult to identify individual cells. IL-36Ra reduced neutrophil presence in both injured ND and injured HFD mice. IR injury did not increase neutrophil adhesion in DR mice to the same degree as in HFD mice with numbers significantly lower than injured HFD mice. Surprisingly, neutrophil adhesion in injured DR mice was also lower than that observed in injured ND mice (Figs. 4a, b and Figure S2). The greatest platelet presence was also identified in injured HFD mice with aggregates again observed primarily in coronary capillaries. Neither IL-36Ra nor DR modified platelet presence in injured mice (Figs. 4a, c and Figure S2).

Since the enzymes that can amplify the biological activity of IL-36 can be bound to NETs expelled from activated neutrophils, NETs were imaged intravitally. No basal difference in their presence was observed between all sham mice but. However, NET formation increased significantly in ND and HFD mice in response to injury, more so in the latter where they were often identified as clusters. IL-36Ra significantly reduced NETs in injured ND and injured HFD mice. IR injury did not increase NET formation in DR mice to the same degree as in HFD mice. Indeed, their presence was significantly lower in injured DR mice compared to injured HFD mice and also, surprisingly, when compared to injured ND mice (Fig. 4d, e). Since IL-36 cytokines are only released after cellular necrosis, intravital imaging was also performed to image necrosis in the beating heart. Whilst no difference in PI-detectable cellular necrosis was observed intravitally between all sham mice, this event increased significantly in injured ND and injured HFD mice and more so in the latter. IL-36Ra significantly reduced cellular necrosis in both injured ND and HFD mice. Again, IR injury did not increase cellular necrosis in DR mice to the same degree as in HFD mice, with levels comparable to injured ND mice (Fig. 4f–g).

### HFD decreases myocardial perfusion—alleviated with IL-36Ra and DR

**Fig. 5:**
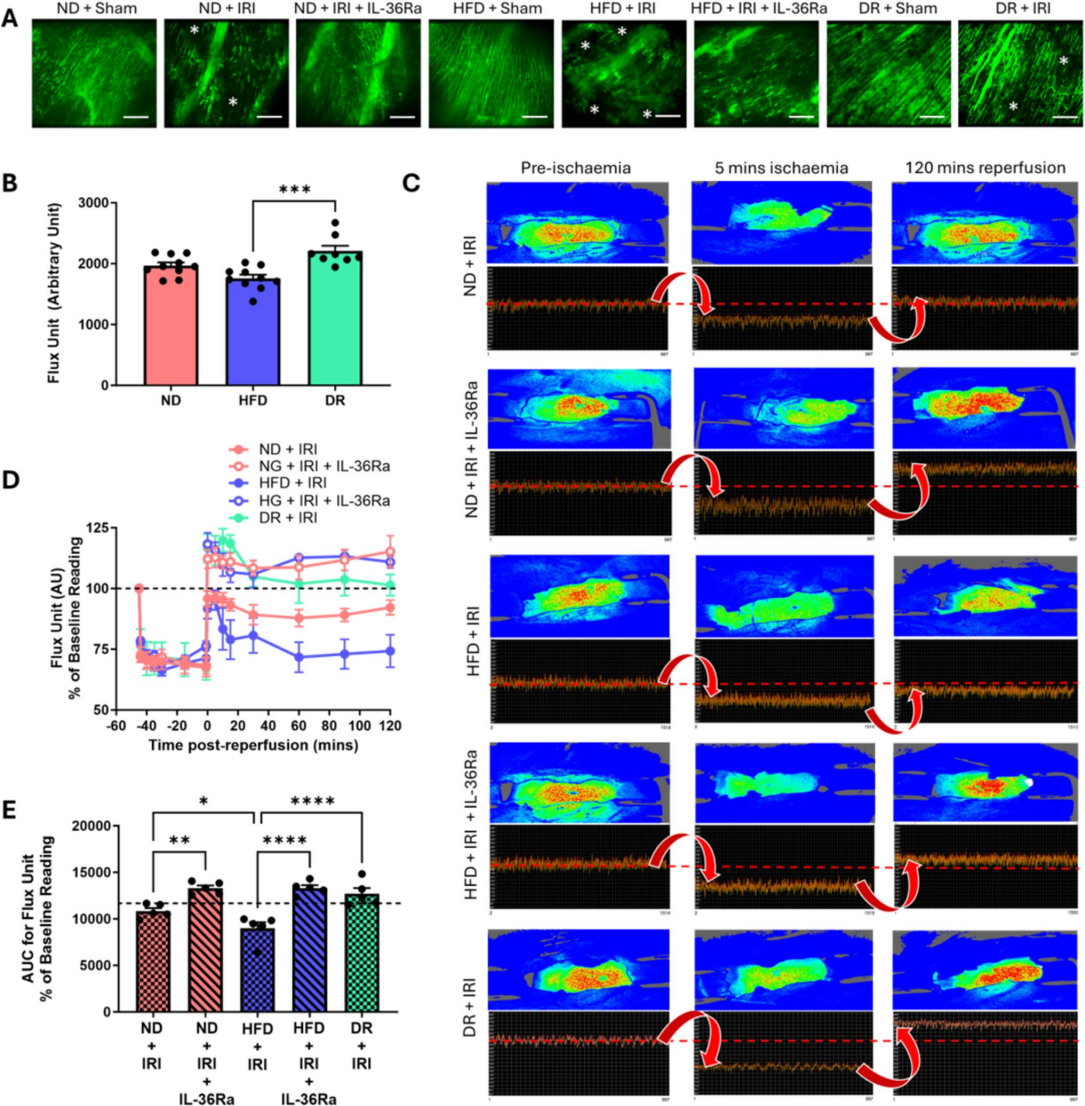
Impact of HFD, IL-36Ra and DR on ventricular perfusion in vivo. **A** Representative intravital images showing FITC-BSA perfused coronary microvessels at 120 min post-reperfusion. Patchy black areas devoid of perfusion are indicated by the asterisk. N = 3/group. **B** Quantitative analysis of baseline flux unit (perfusion) readings obtained using LSCI prior to IR injury. N = 8–10/group **C** Representative LSCI heatmaps and corresponding 1-min real-time flux readouts taken of the beating heart. Heatmaps show warm colours under basal conditions, cooler colours during ischaemia and warmer colours again as reperfusion is initiated. **D**, **E** Quantitative time-course analysis of flux unit readings as a percentage of baseline values obtained by LSCI during the reperfusion phase only and corresponding area under the curve (AUC). The dotted line indicates baseline flux prior to injury with values below and above the line indicative of reduced and increased perfusion respectively. N = 5 mice/group. *p < 0.05, **p < 0.01, ***p < 0.001, ****p < 0.0001 when tested using a one-way ANOVA followed by a Tukey multiple comparison test. ND = normal diet; HFD = high fat diet; DR = diet reversal. Scale bar: 100 μm

Thromboinflammatory cell adhesion, NET release and vascular leakage can collectively hinder the passage of blood through capillaries. Therefore, blood perfusion was assessed intravitally and using LSCI. An extensive network of FITC-BSA perfused capillaries was observed intravitally in all sham mice with well-perfused medium-sized vessels also evident. IR injury resulted in an increase in patchy areas devoid of FITC-BSA perfusion. Qualitatively, the reduced presence of FITC-BSA, indicative of diminished FCD, was more pronounced in injured HFD mice compared to injured ND mice. IL-36Ra treatment improved FCD although some areas lacking capillary perfusion were still discernible. IR injury did not decrease FCD in DR mice to the same degree as in HFD mice (Fig. 5a).

No difference in baseline flux values, indicative of perfusion, was observed using LSCI between uninjured ND and HFD mice, although DR mice had significantly higher baseline perfusion when compared with HFD mice (Fig. 5b). As expected, LSCI heatmaps showed a rapid and striking transition to cooler colours as soon as the LAD artery ligation was performed in all mice, with concomitant decreases in the flux values. The heatmap colour and flux values failed to recover to baseline values in ND and HFD mice once the LAD artery ligature was removed. However, perfusion was significantly poorer in injured HFD mice when compared to ND mice. IL-36Ra improved overall ventricular perfusion, restoring it to similar levels in both ND and HFD mice. The restoration of blood flow was successful in DR mice. Indeed, in all IL-36Ra treated mice and DR mice, reperfusion was accompanied by a hyperaemic response immediately post-reperfusion, which plateaued but remained above baseline values at all time points (Fig. 5b–e).

### HFD increases infarct size, VCAM- 1 and oxidative damage—alleviated with IL-36Ra and DR

**Fig. 6:**
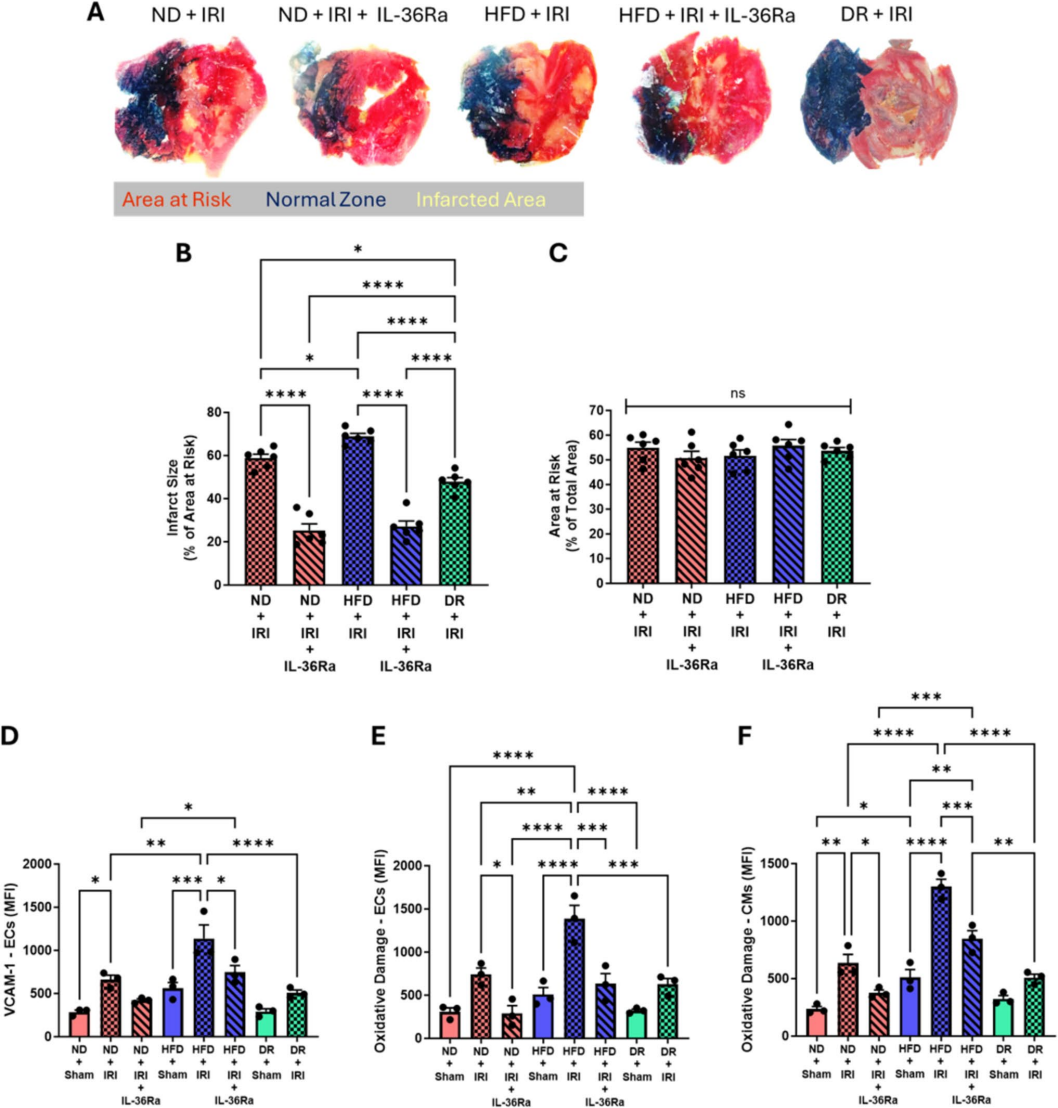
Impact of a HFD, IL-36Ra and diet reversal on infarct size, VCAM- 1 expression and oxidative damage. **A** Representative images of dual Evan’s blue and TTC-stained heart sections to show infarcts. N = 6/group. **B**, **C** Quantitative analysis of infarct size and area at risk (AAR). Flow cytometric analysis for **D** VCAM- 1 expression **E** oxidative damage on ECs and **F** oxidative damage on CMs. N = 3/group. *p < 0.05, **p < 0.01, ***p < 0.001, ****p < 0.0001 when tested using a one-way ANOVA followed by a Tukey multiple comparison test. *ND* normal diet, *HFD* high fat diet, *DR* diet reversal

Infarct size was significantly larger in injured HFD mice compared to injured ND mice. IL-36Ra significantly decreased infarct size in ND and HFD mice to similar levels in both groups. The infarct size in DR mice was significantly less than that observed in both injured ND and HFD mice but was significantly greater than IL-36Ra mice. The area at risk showed no significant differences across all groups (Fig. 6a–c). No basal difference in VCAM- 1 expression was observed flow cytometrically between all sham mice but expression significantly increased in ND and HFD in response to injury and more so in the latter. IL-36Ra decreased VCAM- 1 in injured ND and HFD mice. IR injury did not increase VCAM- 1 in DR mice to the same degree as in HFD mice (Fig. 6d). Similar observations were made when flow cytometrically analysing endothelial cells and cardiomyocytes for oxidative damage (Fig. 6e, f).

### HFD increases serum inflammatory cytokines—alleviated with IL-36Ra and DR

Multiplex analysis demonstrated HFD alone significantly increased the basal circulating levels of 13 of 23 inflammatory cytokines tested compared to ND mice. 14 cytokines increased significantly in injured ND mice when compared to sham ND mice. 13 cytokines increased significantly in injured HFD mice when compared to sham HFD mice. IL-36Ra significantly decreased 13 cytokines in injured ND mice compared to untreated ND mice and 16 cytokines in injured HFD mice compared to untreated HFD mice. Notably, IL-4 and IL-13 were the only two cytokines significantly elevated in IL-36Ra treated injured ND and HFD mice. DR mice did not demonstrate the similar increases in circulating cytokines that were noted in HFD mice. Indeed, 14 cytokines significantly decreased in DR sham mice compared to HFD sham mice and 16 cytokines significantly decreased in injured DR mice compared to injured HFD mice. Surprisingly, 14 cytokines were even significantly lower than injured ND mice. IL-13 was the only cytokine that significantly increased in injured DR mice compared to injured HFD mice with the highest circulating levels of IL-13 detected in injured DR mice (Table 1 and Figure S3).

**Table 1:**
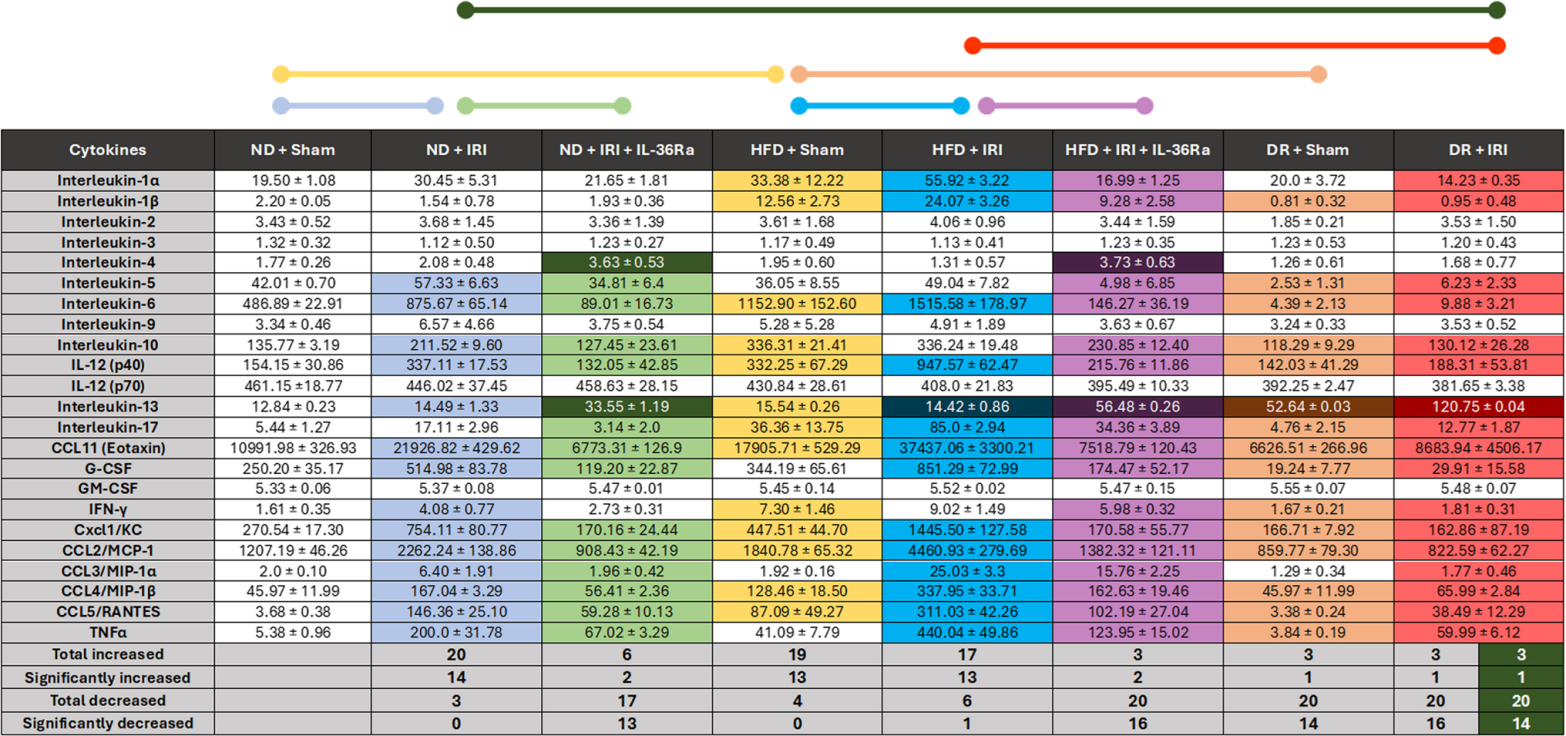
Impact of a HFD, IL-36Ra and diet reversal on circulating serum levels of inflammatory cytokines

Multiplex ELISA was used to assess cytokine levels. Statistical comparisons made are shown above the table. For example, the red line indicates that when the HFD + IRI group were compared to the DR + IRI group, the cytokines shaded in red in the individual cells were significantly different. Coloured cells indicate comparisons that were significantly different. The darker shading of the same colour indicates cytokines that increased or decreased in the opposite direction to the overall changes. Results are presented as mean ± SEM pg/ml. N = 3/group

*ND* normal diet, *HFD* high fat diet, *DR* diet reversal

## Discussion

This study provides original insights into the behaviour of the coronary microcirculation in mice fed a hyperglycaemia-inducing HFD, and how its response to IR injury differs from normoglycaemic hearts in vivo. We show that even basally, multiple microvascular differences existed, including enhanced leakage in response to histamine, altered vasotone and increased neutrophil and platelet presence. Greater cardiac expression of IL-36/IL-36R and raised levels of circulating pro-inflammatory cytokines were also observed. Moreover, in the immediate aftermath of reperfusion, a HFD was associated with significantly enhanced thromboinflammatory responses, greater necrotic cell and NET presence and poorer overall ventricular perfusion, ultimately leading to larger infarcts. A new mechanistic role for the IL-36/IL-36R signalling pathway is described for the first time in this co-morbidity model of MI, inhibition of which reassuringly remained vasculoprotective even in the highly inflamed hearts of injured HFD mice. Switching back to a ND could also mitigate the heightened microcirculatory perturbations associated with a HFD following IR injury. We postulate that these benefits may have been partly exerted by initiating a compensatory or adaptive systemic anti-inflammatory response mediated by the related IL-4 and IL-13 cytokines.

Diabetes is known to cause microvascular dysfunction in a number of vascular beds, but less is known about its direct effects on coronary microvessels in vivo. Here we show that the impact of an HFD-induced chronic hyperglycaemia on the coronary microcirculation was not negligible. Although leaky capillaries are a well described complication of diabetic retinopathy [50], our study is the first to directly visualise enhanced leakage from coronary capillaries in vivo in response to topical histamine in hyperglycaemic mice compared to healthy hearts. Interestingly, mast cells neighbouring coronary microvessels are not only more abundant, but also degranulate more readily, in the diabetic heart [23]. This could potentially pose a significant problem when considering mast cells are the major stored source of histamine. Another novel aspect of our work was the assessment of microvascular tone in the beating heart in vivo*.* Attenuated vasoconstriction but increased vasodilation responses were observed in medium-sized blood vessels in HFD mice. This differs from the well-recognised impairment of both observed clinically and experimentally in diabetic coronary artery and non-coronary vessels [29, 33, 45]. It is possible that a longer duration of hyperglycaemia is required before vasodilatory dysfunction becomes apparent in our model. Indeed, early and uncomplicated diabetes is accompanied by arterial and venular dilation, possibly due to increased nitric oxide production, presaging increased capillary permeability and their eventual destruction [62]. In the current study, it was difficult to ascertain whether vasotonal changes occurred in arteries/arterioles or veins/venules. Within the cremaster muscle, most commonly imaged intravitally, these two vessel types are easy to distinguish due to its transparent tissue nature. Cremasteric arteries/arterioles can be identified by their direction of flow, which is faster and pulsatile. Also, these vessels are more rounded, darker in appearance and have an absence of rolling leukocytes. However, in the beating heart, these discerning features were not as obvious due to the relatively poorer image resolution associated with imaging a solid and moving organ that has much smaller blood vessels in the field of view than those imaged in the cremaster. Since arteries/arterioles generally tend to have stronger vasoconstrictor and vasodilator responses to vasoactive substances, our observed responses may be occurring in these vessels, but confirmation would require further investigation.

Neutrophil functions, such as migration and phagocytic ability, are commonly dysregulated in T2DM, which may explain the weakened response to infection in patients with diabetes. However, increased neutrophil and endothelial adhesion molecule expression, and decreased neutrophil deformability, has also been described, which may explain the almost twofold increased basal presence of neutrophils in the current study [9, 49]. We noted a number of pro-inflammatory neutrophil pathways also raised as a consequence of hyperglycaemia including NET formation, ROS generation and cytokine generation, all of which can damage the local microvascular environment and perpetuate inflammation [9]. Indeed, the potently pro-inflammatory IL-1α, IL-1β, IL-6 and IFNγ cytokines were all raised basally in HFD-fed mice. Presence of platelet microaggregates was also higher, accompanied by a remarkable basal activation of platelets as evidenced by increased P-selectin expression (data not presented). This is consistent with findings in patients and animal models of T2DM where increased baseline platelet activation and hyperreactivity has been observed following acute and chronic hyperglycaemia [26]. These basal microcirculatory disturbances and low-grade systemic inflammation, when coupled with an acute MI insult, could synergistically amplify coronary perturbations in the setting of diabetes.

Indeed, this study offers the first direct demonstration of the rapid and devastating impact of reperfusion on the smallest blood vessels of the heart in the setting of hyperglycaemia in vivo. Remarkable neutrophil adhesion was observed within coronary capillaries in injured HFD mice. They frequently appeared as clusters that occupied entire capillaries, making it challenging to delineate individual cells. Supporting previous studies [14], we also showed increased ROS specific damage of coronary ECs and CMs and increased endothelial VCAM- 1 expression in injured HFD mice, which likely contributed mechanistically to enhancing neutrophil adhesion. Increased platelet presence, platelet activation and circulating platelet counts were also highest in these mice, although their coronary recruitment was not as remarkable as neutrophils. Consequently, these thromboinflammatory occlusive events resulted in the poorest ventricular perfusion in the injured hearts of HFD mice, evidenced intravitally by patchy areas devoid of FITC-BSA. Cellular necrosis was also imaged for the first time in the beating heart in vivo and was most prominent in flow-devoid regions. Since PI, used to detect necrosis, was injected systemically, it most likely detected EC death. However, it would have been able to leak through damaged vessels and also label necrotic cardiomyocytes, confirmation of which would require further investigation.

Our improved stabilisation method, combined with high magnification imaging and triple labelling, permitted direct visualisation of NETs for the first time in the beating heart in vivo. Elevated NET presence was observed in injured HFD mice, particularly in areas where SYTOX™ green staining identified a high number of dead cells. However, NET presence did not match the extensive presence of neutrophils, indicating that not all neutrophils were prone to release NETs. Interestingly, they did not show the typical morphology of web-like threads, appearing more as round or oval clumps which we speculate may have increased their potential to be vascular occlusive. Indeed, NETs have been shown to play an aggravating pathological role in sterile injuries, promoting vascular occlusion by providing a scaffold for trapping RBCs and platelets [16, 47, 63]. In diabetic mice, circulating NETs have also been described as important disruptors of macrovascular function, specifically impairing vasodilation [39]. Increased circulating NET markers and NET presence in thrombi aspirated from occluded coronary arteries has also been found in MI patients, which positively correlated with infarct size [19, 43].

We previously demonstrated enhanced expression of IL-36 and its receptor in the heart and coronary ECs of aged mice [10] and now describe a similar up-regulation following chronic hyperglycaemia in HFD mice. In both aged and HFD-fed mice, neutrophil recruitment was a key basal feature which was enhanced further after injury. Interestingly, this inflammatory response appeared to be greater in aged mice, as was the response to topical IL-36 exposure. Indeed, remarkable neutrophil adhesion occurred in both capillaries and larger blood vessels in aged mice but only capillaries were predominantly involved in HFD-fed mice [10]. Noteworthy was the greater thrombotic presence in the coronary microcirculation of injured hyperglycaemic mice compared to aged mice. Despite these subtle differences, IL-36Ra was equally potent at reducing infarct size in both mice. This alludes to the possibility that the chronic low-grade inflammation or ‘metainflammation’ associated with hyperglycaemia shares the same mechanisms underpinning ‘inflammaging’ in the heart. In the current study, IL-36Ra was vasculoprotective in all injured hearts, reducing neutrophil recruitment and consequently improving FCD and overall ventricular perfusion. Mechanistically, these benefits could be attributed to the reduction in cellular necrosis, reduced endothelial VCAM-1 expression and/or reduced oxidative damage. NET presence was also decreased, and whilst this may simply have been due to decreased numbers of recruited neutrophils, it is possible that neutrophils in IL-36Ra treated mice were less capable of generating NETs. Indeed, this has been shown when neutrophils have been treated in vitro with anakinra, an IL-1 receptor antagonist [59].

IL-36 cytokines are potent pro-inflammatory factors that are not known to directly activate and aggregate platelets. Indeed, the IL-36R has not been identified on platelets as far as we are aware. However, similar to IL-1 cytokines, they may mediate platelet activation indirectly through inflammation-driven thrombosis. However, the lack of any therapeutic effect on platelets in the current study suggests inflammation may not be key in driving platelet recruitment. This suggests it likely occurs after direct platelet activation e.g. platelets interacting with sub-endothelial matrix proteins such as collagen exposed after endothelial denudation. Larger infarct size is well described in experimental models of T2DM, confirming our own results [8]. Therefore, it was encouraging to show that the IL-36Ra mediated vasculoprotection reduced infarct size to similar levels in both ND and HFD mice despite the larger infarcts in the latter.

Multiplex analysis demonstrated that 83% of the tested cytokines were increased basally as a consequence of a 16 week HFD, supporting the prominent chronic low grade systemic (meta)inflammation feature of T2DM. Indeed, IL-1β, IFNγ, TNFα and IL-6, amongst the key cytokines implicated in diabetic inflammation of many organs [57], showed the most remarkable increases in HFD mice compared to ND mice. Although not usually associated with any notable structural changes or loss of tissue function, mild inflammatory and immune cell infiltration can occur basally and was indeed noted in the coronary microcirculation of HFD mice in the current study. However, an acute IR injury further increased the levels of circulating cytokine and chemokine levels in both ND and HFD mice, with IL-1α/β, IL-6, IL-12, IL-17 and TNFα reaching their highest levels in the latter group. It is worth stating that the surgical procedure may have increased the levels of circulating cytokines as has been previously described, and may also have exaggerated the subsequent increase in response to injury [48]. Nevertheless, our data indicates a highly pro-inflammatory systemic environment in these mice, consistent with previous findings in patients and animal models [25]. Collectively, these can directly or indirectly mediate any one of the microcirculatory perturbations we observed in the injured HFD mice.

Persistent inflammation is known to concomitantly stimulate innate compensatory anti-inflammatory responses to prevent excessive tissue damage [51, 53]. Indeed, elevated levels of IL-1Ra, and known anti-inflammatory cytokines IL-10, IL-4 and IL-13, have been described either preceding or during the development of T2DM [20]. Indeed, we also noted elevated levels of IL-10 in response to IR injury and hyperglycaemia. Hence, this master anti-inflammatory cytokine may contribute to some of the therapeutic effects observed in this study. Whilst the related cytokines IL-4 and IL-13 are often thought of as pro-inflammatory in type 2 inflammatory conditions, they can also be anti-inflammatory through an ability to suppress inflammation by downregulating IL-1β and TNFα production by fibroblasts and endothelial cells [3, 35]. To consolidate these differing opinions, it has been speculated that they may exert a pathological role in the early stages of a disease but an inhibitory effect when the disease is more established [24]. In the current study, novel data showed that although the secretory profile for IL-4 did not generally change, significant increases to its highest levels were observed in IL-36Ra treated injured ND and HFD mice. IL-13 levels increased in response to HFD and IR injury but, similar to IL-4, also increased further in response to IL-36Ra treatment. However, a > eightfold increase in IL-3 was observed in IR injured HFD mice that had been switched back to a ND. Moreover, these observations were unique to IL-4 and IL-13. Interestingly, Sun and colleagues also noted increased levels of IL-4 and IL-13 in mice switched from being fed the same HFD for 11 weeks to normal chow [56]. The fact that we showed their greatest levels in mice in which a pharmacological or dietary therapeutic strategy had been implemented was surprising. This suggests IL-36Ra or dietary modification may use similar endogenous anti-inflammatory pathways as part of their beneficial mechanism and raises the possibility of exploiting the signalling pathways of these related cytokines as alternative vasculoprotective strategies in T2DM. Indeed, a salient finding of this study was the observation that IR injury had less of an effect on neutrophil recruitment and NET formation in injured DR hearts compared to injured ND hearts, which may be explained by these raised IL-4/IL-13 levels. However, it is important to note that IL-36Ra is a pure receptor antagonist and, so far, has no known biological activity other than to block IL-36 cell surface receptors. Therefore, it is not clear why the use of this therapy in our hands increased IL-4/IL-13 activity. Interestingly, anti-TNF therapy has been shown to be therapeutic by a mechanism that increases anti-inflammatory IL-10 synthesis [31]. Whether such unknown therapeutic mechanisms of action also exist for IL-36Ra requires further investigation.

To test whether our observed heightened susceptibility of the coronary microcirculation to IR injury was permanent or reversible after normalising the chronic hyperglycaemia, we imaged the beating hearts of HFD mice that had been switched to a ND. Whilst the HFD was accompanied by weight gain and plasma glucose levels exceeding 250 mg/dl, a value considered diabetic in rodents, DR resulted in significant weight loss and plasma glucose levels < 200 mg/dl, considered normal in rodents [13]. Consequently, coronary microvessels did not demonstrate the exacerbated leakage in response to histamine nor the disturbed vascular tone. Furthermore, basal platelet presence in coronary microvessels and systemic platelet activation was also reduced. This indicates for the first time that basal coronary microvessel damage, as a result of previously being chronically hyperglycaemic, is not permanent. Moreover, in response to injury, the perturbed neutrophil adhesion, FCD and ventricular perfusion were also alleviated. Mechanistically, this could be due to reduced NET formation, cellular necrosis, oxidative damage, VCAM-1 expression and also IL-36R expression. Hence, we propose that DR utilises comparable protective mechanisms to those used by IL-36Ra.

### Limitations of the study

We recognise that stabilisation methods to reduce motion within the heart can potentially induce tissue damage or alter coronary physiology. Indeed, it is a fine line between balancing tissue immobilization for high-resolution imaging while maintaining physiological function and minimizing trauma. To circumvent this, only the region within the immediate centre of the stabilisers used was imaged rather than near the edges of the stabiliser ring. Whilst coronary perturbations such as leakage, absence of flow and inflammatory cell infiltration were minimal in sham mice in this central area, we cannot exclude the possibility of some interference at a sub-microscopic level. An additional limitation of this study was the testing of only a single dose of ACh and PE to assess vasotonal responses. Whilst changes in microvessel calibre were indeed observed with the doses used, without investigating dose–response effects, we cannot conclude whether maximal vasodilation/vasoconstriction occurred and whether partial, subtle or severe endothelial dysfunction had been impacted in the chronically hyperglycaemic mice. This study also focused on the role of inflammation in mediating the various coronary microcirculatory perturbations we described. However, it is important to note that coronary flow is also influenced by metabolic rate, myogenic forces, extravascular compression, oedema and neurohumoral agents. Therefore, an important limitation of this study is that it did not investigate the extent to which each of these, if any, could also have contributed to the observed responses in the setting of chronic hyperglycaemia. A detailed intra and interobserver variability analysis has also not been performed and therefore we acknowledge this as a limitation of the study. Exploring sex-related differences was beyond the scope of this already multifaceted study. This must be addressed in future studies in light of our own research that did indeed report an increased inflammatory cell presence in IR injured coronary microvessels in female mice when compared to male mice. However, this heightened inflammatory response in female mice could still be alleviated with the use of IL-36Ra therapy [12]. This suggests this strategy has the potential to achieve a similar benefit in diabetic female mice if tested.

## Concluding remarks

A functioning coronary microcirculation is imperative in order to improve long-term patient outcomes, particularly in MI patients with diabetes where no-reflow is a frequent occurrence [64]. We propose that inhibiting the receptor for the pro-inflammatory IL-36 cytokine in these co-morbid patients may offer coronary vasculoprotective benefits and reduce infarct size. This is an exciting time for IL-36 research in the setting of this co-morbidity. Whilst we show that inhibiting IL-36 benefits the highly perturbed hyperglycaemic injured coronary microcirculation, others have recently shown that inhibition of IL-36 can alleviate insulin resistance in HFD-fed obese mice with diabetes [40, 61]. Importantly we show that dietary modification can mitigate the heightened microcirculatory perturbations associated with a HFD, potentially through a compensatory anti-inflammatory response. These novel findings are the first to highlight the specific benefits of reversing chronic hyperglycaemia on the coronary microcirculation and possibly even enhancing coronary resilience in the event of a subsequent MI. Furthermore, our novel data from the DR studies uncover additional mechanisms involving the related IL-4 and IL-13 cytokines that could be tested for their vasculoprotective efficacy in future studies.

## Supplementary Information

Below is the link to the electronic supplementary material.

Supplementary file1 (DOCX 472 KB)

## Data Availability

The authors confirm that the data supporting the findings of this study are available within the article and its supplementary materials. Raw data supporting the findings of this study are available from the corresponding author, Dr Neena Kalia, on request.
