# Supplementary material for: Impact of chronic hyperglycaemia on the coronary microcirculation – benefits of targeting IL-36 and diet reversal
Source: Basic Res Cardiol. 2025 Apr 17;120(3):509–26. doi: 10.1007/s00395-025-01107-y (PMC12158858; doi:10.1007/s00395-025-01107-y)
Supplement: Supplementary file 1 — Supplementary file1 (DOCX 472 KB) [file 395_2025_1107_MOESM1_ESM.docx]

**Supplementary Figure – Figure S1**

**
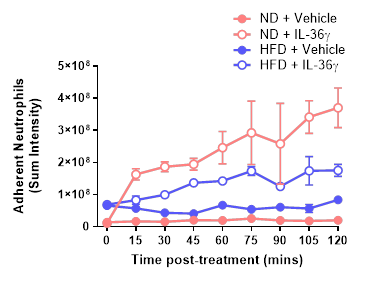
**

**A**

**
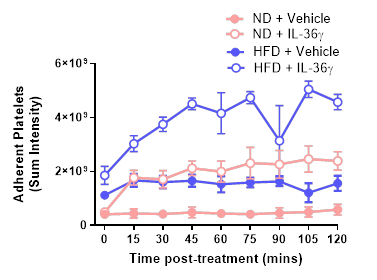
B**

**Figure S1. Neutrophil and platelet recruitment in response to topical exposure of the beating heart coronary microcirculation to IL-36 *in vivo***. Quantitative analysis of the intravital data for adherent **(A)** neutrophils and **(B)** platelets to illustrate the dynamics of their recruitment after topical exposure to IL-36γ over an imaged period of 2 hours. Recruitment of these cells at the single time point of 120 minutes, and the statistical analysis of this data, is available in **Figure 3H** and **Figure 3I** of the main text. N=3/group. ND = normal diet ; HFD = high fat diet.

**Supplementary Figure – Figure S2**


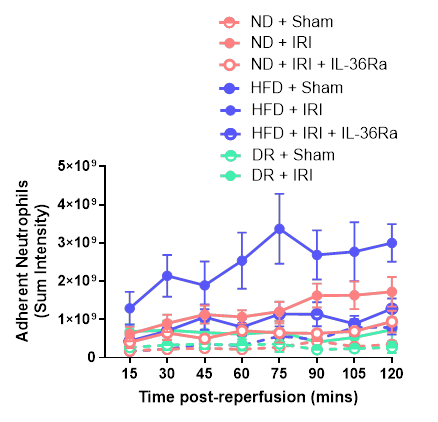
**A B**


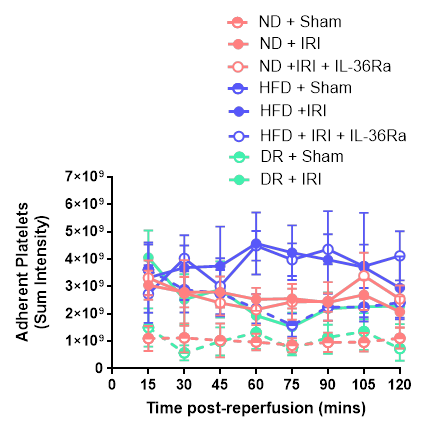


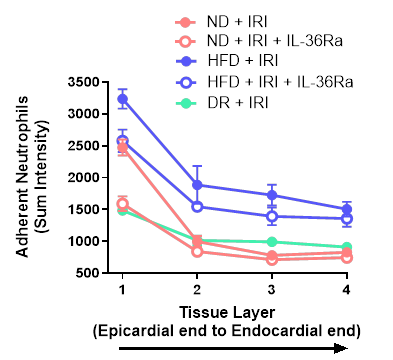
**C**

**Figure S2. Impact of a high fat diet, IL-36Ra therapy and diet reversal on neutrophil and platelet recruitment in the IR injured beating heart coronary microcirculation *in vivo*.** Quantitative analysis of intravital data for adherent **(A)** neutrophils and **(B)** platelets to illustrate the dynamics of their recruitment over an imaged reperfusion period of 2 hours. Recruitment of these cells at the single time point of 120 minutes, and the statistical analysis of this data, is available in **Figure 4B** and **Figure 4C** of the main text. **(C)** Multiphoton data for adherent neutrophils at various depths of the left ventricle (LV). To assess whether intravital observations from the heart surface were mirrored throughout the thickness of the ventricular wall, multiphoton microscopy was performed on hearts harvested at the end of intravital experimentation as previously described [10]. Briefly, the LV was vibratome-cut into four 300µm sections and multiphoton z-stacks were obtained from all four layers (FVMPE-RS Olympus). . N=5-6/group. ND = normal diet ; HFD = high fat diet ; DR = diet reversal.


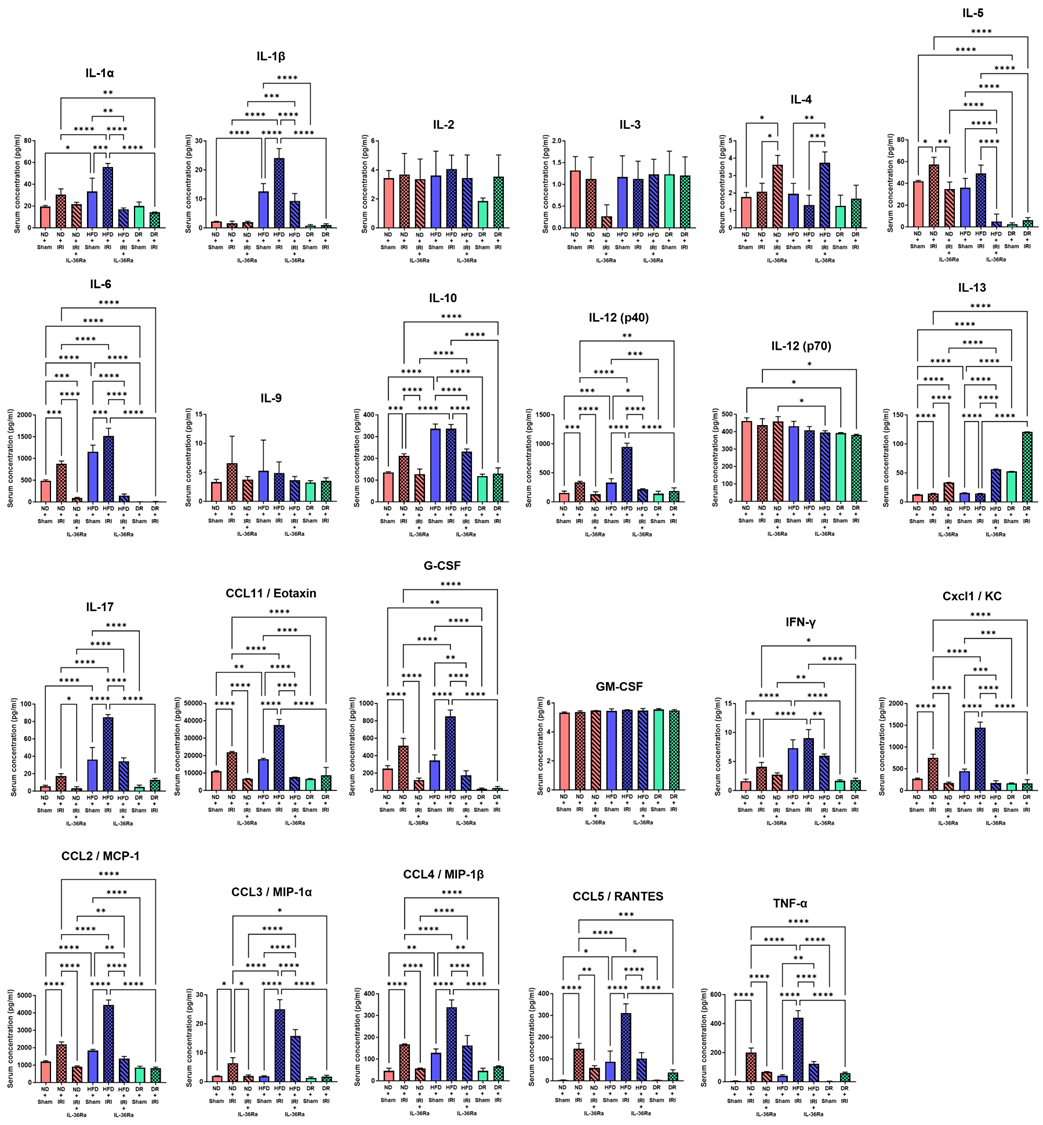
**Supplementary Figure – Figure S3**

**Figure S3. Impact of a high fat diet, IL-36Ra therapy and diet reversal on circulating serum levels of 23 inflammatory cytokines.** Multiplex ELISA was used to assess inflammatory cytokines levels. A tabled version of this data, summarising the key significant differences, is available in **Figure 8** of the main text. N=3/group. *p<0.05, **p<0.01, ***p<0.001, ****p<0.0001 when tested using a one-way ANOVA followed by a Tukey multiple comparison test. ND = normal diet ; HFD = high fat diet ; DR = diet reversal.
